# Supplementary material for: Insights and caveats from mining local and global temporal motifs in cryptocurrency transaction networks
Source: Sci Rep. 2024 Nov 4;14:26569. doi: 10.1038/s41598-024-75348-7 (PMC11535018; doi:10.1038/s41598-024-75348-7)
Supplement: Supplementary file 1 — Supplementary Information. [file 41598_2024_75348_MOESM1_ESM.pdf]

Supplementary information for “Mining local and global temporal motifs from cryptocurrency transaction networks”

September 13, 2024

1 Motifs arising from transactions in the same block

In a blockchain setting, in particular for Bitcoin and Ethereum which are the cryptocurrencies used in both our datasets, transactions are released in *blocks* according to when they are successfully verified by a miner, typically in time intervals of around 8-10 minutes. This means that while a transaction can be initiated by a user/service at any continuous time, it is only possible to observe the block-wide timestamp of each timestamp. In particular, this means that many transactions receive the same timestamp. Since the motifs involve an ordering on the transactions involved, the method for deciding an ordering will affect the motif counts. For example, consider the sequence of transactions $\tau_1 = (A, B, t_1)$, $\tau_2 = (B, C, t_2)$, $\tau_3 = (C, A, t_2)$ with $t_1 < t_2$. Both τ_2 and τ_3 have the same timestamp. If we assume τ_2 occurred before τ_3 then these motifs would be an instance of motif $M_{2,4}$, a time-respecting cyclic triangle. If instead we placed τ_3 before τ_2, then it would be an instance of $M_{3,5}$ a cyclic triangle that is no longer time-respecting.

One approach would be simply to enforce that transactions forming a motif have all different timestamps. However, this would result in a large information loss given that transactions happening at the same time is a feature of these networks rather than an anomaly. Another approach we considered was to tie-break events with the same timestamp by other ordered attributes such as the transaction's sender node ID then receiver ID. This would have the benefit of being robust to the order in which the transactions were being read in, but had the potential to introduce bias (the transactions $\tau_1 = (A, B, t_1)$, $\tau_2 = (B, C, t_2)$, $\tau_3 = (C, A, t_2)$ would always be counted as motif $M_{2,4}$ regardless of the true order of τ_2 and τ_3 for example). We chose instead to order additionally by the order the transaction appears in the block (in this case corresponding to the file line number). This is not guaranteed to be the correct time order but transactions earlier in the block are likely to be before those later in the block. Specifically, if a transaction τ_2 spends an output of transaction τ_1, then τ_2 is always placed after τ_1 in the block.

Nonetheless, to test the robustness of this choice of ordering, we performed an experiment to test how much the motif counts vary over 10 different randomisations of intra-block transaction orders. We produced 10 files with transactions having same timestamp randomised and performed the motif counts on each of them. Fig. 1 shows the coefficient of variation over these 10 experiments. At worst, some of the triangle motifs (whose counts were relatively very low in this dataset) have a standard deviation of around 3% of their mean value, and most motifs have much lower variation. $M_{6,1}$, the two-node one-direction motif has a value of 0 as there is no ambiguity in how to interpret multiple same-direction transactions between the same pair of nodes.

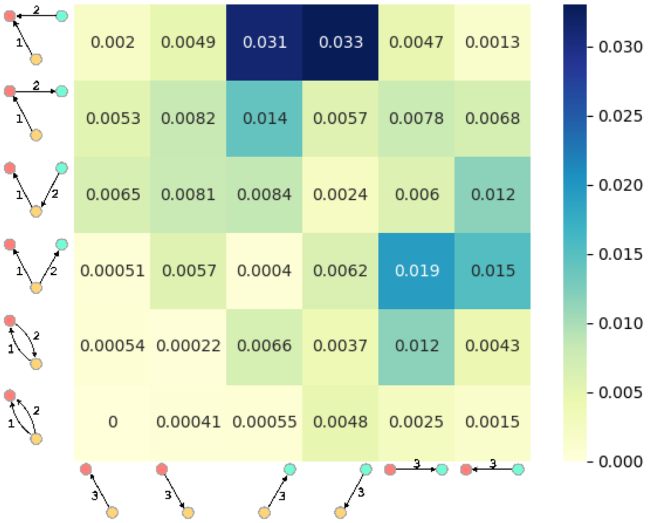

Figure 1: Experiment to test robustness of tie-breaking method for transactions within the same block, tested on Alphabay transactions with $\delta = 1$ hour. Shown is the coefficient of variation (standard deviation divided by the mean value) for each motif, calculated over 10 experiments.
